# Supplementary material for: Analysis of human mitochondrial genome co-occurrence networks of Asian population at varying altitudes
Source: Sci Rep. 2021 Jan 8;11:133. doi: 10.1038/s41598-020-80271-8 (PMC7794584; doi:10.1038/s41598-020-80271-8)
Supplement: Supplementary file 1 — Supplementary Information. [file 41598_2020_80271_MOESM1_ESM.doc]

Supplementary Information

Title

**Analysis of human mitochondrial genome co-occurrence networks of Asian population at varying altitudes**

Authors

**Rahul K Verma^1^, Alena Kalyakulina^2^, Cristina Giuliani^3^, Pramod Shinde^4^, Ajay Deep Kachhvah^5^, Mikhail Ivanchenko^2,6^, and Sarika Jalan^1,5,6,7^**

1. Discipline of Biosciences and Biomedical Engineering, Indian Institute of Technology Indore, Khandwa Road, Simrol, Indore-453552, India

2. Department of Applied Mathematics and Centre of Bioinformatics, Lobachevsky State University of Nizhny Novgorod, Nizhny Novgorod, Russia

3. Laboratory of Molecular Anthropology & Center for Genome Biology, Department of Biological, Geological and Environmental Sciences, University of Bologna, Italy

4. Division of Vaccine Discovery, La Jolla Institute for Immunology, La Jolla, CA 92037, United States.

5.Complex Systems Lab, Discipline of Physics, Indian Institute of Technology Indore, Khandwa Road, Simrol, Indore-453552, India

6.Laboratory of Systems Medicine of Healthy Aging and Department of Applied Mathematics, Lobachevsky University, Nizhny Novgorod, Russia 7. Center for Theoretical Physics of Complex Systems, Institute for Basic Science (IBS), Daejeon, 34126, Republic of Korea

**Corresponding Author:** Sarika Jalan
**Email address:** sarika@iiti.ac.in

**Altitude range: 0-500 m**

| Accession number | Haplogroup | Country | Reference |
| --- | --- | --- | --- |
| KX467277.1 | NG187 | India | https://doi.org/10.1038/s41598-017-18893-8 |
| KX467263.1 | NG33 | India | https://doi.org/10.1038/s41598-017-18893-8 |
| KX467306.1 | NG8 | India | https://doi.org/10.1038/s41598-017-18893-8 |
| KX467290.1 | NG17 | India | https://doi.org/10.1038/s41598-017-18893-8 |
| KX467320.1 | NG9 | India | https://doi.org/10.1038/s41598-017-18893-8 |
| KX467321.1 | NG10 | India | https://doi.org/10.1038/s41598-017-18893-8 |
| KC911479.1 | U2e1a1* | Iran | 10.1371/journal.pone.0080673 |
| KC911577.1 | I1a* | Iran | 10.1371/journal.pone.0080673 |
| KC911502.1 | U1a1* | Iran | 10.1371/journal.pone.0080673 |
| KC911457.1 | U1a3b | Iran | 10.1371/journal.pone.0080673 |
| KU178924.1 | NG192 | Iran | 10.1371/journal.pone.0080673 |
| KC911405.1 | U2d2 | Iran | 10.1371/journal.pone.0080673 |
| KU178923.1 | NG178 | Iran | 10.1371/journal.pone.0080673 |
| KC911378.1 | U3b1a1 | Iran | 10.1371/journal.pone.0080673 |
| KC911392.1 | U7a* | Iran | 10.1371/journal.pone.0080673 |
| KX467282.1 | NG190 | India | https://doi.org/10.1038/s41598-017-18893-8 |
| KC911624.1 | T1a1b1 | Iran | 10.1371/journal.pone.0080673 |
| KC911423.1 | T2b* | Iran | 10.1371/journal.pone.0080673 |
| KC911528.1 | T2c1c* | Iran | 10.1371/journal.pone.0080673 |
| KC911542.1 | T2g1 | Iran | 10.1371/journal.pone.0080673 |
| KC911355.1 | N3a | Iran | 10.1371/journal.pone.0080673 |
| KC911309.1 | K1a* | Iran | 10.1371/journal.pone.0080673 |
| KC911550.1 | U3a* | Iran | 10.1371/journal.pone.0080673 |
| KC911569.1 | J1b* | Iran | 10.1371/journal.pone.0080673 |
| KC911496.1 | J1b1b1* | Iran | 10.1371/journal.pone.0080673 |
| KC911356.1 | B4b1a2e | Iran | 10.1371/journal.pone.0080673 |
| KC911341.1 | W* | Iran | 10.1371/journal.pone.0080673 |
| KX467289.1 | NG14 | India | https://doi.org/10.1038/s41598-017-18893-8 |
| KC911561.1 | W* | Iran | 10.1371/journal.pone.0080673 |
| JQ245728.1 | DL244 | UAE | 10.1016/j.ajhg.2011.12.010 |
| KY934478.1 | W6 | Bulgaria | 10.1016/j.ajhg.2011.12.010 |
| KC911390.1 | A4a1* | Iran | 10.1371/journal.pone.0080673 |
| KC911573.1 | N2a1 | Iran | 10.1371/journal.pone.0080673 |
| KC911409.1 | U5a1g1 | Iran | 10.1371/journal.pone.0080673 |
| KC911408.1 | HV2* | Iran | 10.1371/journal.pone.0080673 |
| KC911292.1 | H15* | Iran | 10.1371/journal.pone.0080673 |
| KC911311.1 | HV1* | Iran | 10.1371/journal.pone.0080673 |
| KX467325.1 | NG75 | India | https://doi.org/10.1038/s41598-017-18893-8 |
| KC911330.1 | H5c1* | Iran | 10.1371/journal.pone.0080673 |
| KC911346.1 | H6a1a* | Iran | 10.1371/journal.pone.0080673 |
| KC911439.1 | HV* | Iran | 10.1371/journal.pone.0080673 |
| KC911412.1 | HV18 | Iran | 10.1371/journal.pone.0080673 |
| KC911482.1 | HV12b1 | Iran | 10.1371/journal.pone.0080673 |
| KC911464.1 | H5m | Iran | 10.1371/journal.pone.0080673 |
| KC911282.1 | H66* | Iran | 10.1371/journal.pone.0080673 |
| KC911454.1 | H13b* | Iran | 10.1371/journal.pone.0080673 |

**Altitude range: 501-1000 m**

| Accession number | Haplogroup | Country | Reference |
| --- | --- | --- | --- |
| KC911432.1 | U5a1d2b | Iran | 10.1371/journal.pone.0080673 |
| KC911402.1 | U7a4a1 | Iran | 10.1371/journal.pone.0080673 |
| KC911615.1 | U7a* | Iran | 10.1371/journal.pone.0080673 |
| KC911523.1 | W6 | Iran | 10.1371/journal.pone.0080673 |
| KC911518.1 | W6 | Iran | 10.1371/journal.pone.0080673 |
| KC911446.1 | W6 | Iran | 10.1371/journal.pone.0080673 |
| KC911484.1 | W6 | Iran | 10.1371/journal.pone.0080673 |
| KC911329.1 | T1a1m1 | Iran | 10.1371/journal.pone.0080673 |
| KC911558.1 | T2m | Iran | 10.1371/journal.pone.0080673 |
| KC911359.1 | J1b5c | Iran | 10.1371/journal.pone.0080673 |
| KC911327.1 | H13a2a* | Iran | 10.1371/journal.pone.0080673 |
| KC911332.1 | C4a6 | Iran | 10.1371/journal.pone.0080673 |
| KX467322.1 | NG48 | India | https://doi.org/10.1038/s41598-017-18893-8 |
| KC911278.1 | U7b* | Iran | 10.1371/journal.pone.0080673 |
| JQ245736.1 | NOS84 | Russia | 10.1016/j.ajhg.2011.12.010 |
| JQ245761.1 | PAL1024 | Russia | 10.1016/j.ajhg.2011.12.010 |
| JQ245762.1 | PAL516 | Russia | 10.1016/j.ajhg.2011.12.010 |
| JQ245723.1 | NOS10 | Russia | 10.1016/j.ajhg.2011.12.010 |
| KC911494.1 | R0a2d | Iran | 10.1371/journal.pone.0080673 |
| KC911453.1 | U4c1a | Iran | 10.1371/journal.pone.0080673 |
| KC911301.1 | HV12b1 | Iran | 10.1371/journal.pone.0080673 |
| KC911476.1 | HV5 | Iran | 10.1371/journal.pone.0080673 |
| KC911576.1 | U5a1* | Iran | 10.1371/journal.pone.0080673 |
| KC911564.1 | U5b2a1a2 | Iran | 10.1371/journal.pone.0080673 |
| KX467307.1 | NG28 | India | https://doi.org/10.1038/s41598-017-18893-8 |
| KX467303.1 | NG129 | India | https://doi.org/10.1038/s41598-017-18893-8 |
| KX467265.1 | NG202 | India | https://doi.org/10.1038/s41598-017-18893-8 |
| KX467316.1 | NG15 | India | https://doi.org/10.1038/s41598-017-18893-8 |
| KX467318.1 | NG69 | India | https://doi.org/10.1038/s41598-017-18893-8 |
| KX467315.1 | NG42 | India | https://doi.org/10.1038/s41598-017-18893-8 |
| KX467319.1 | NG209 | India | https://doi.org/10.1038/s41598-017-18893-8 |
| KX467304.1 | NG51 | India | https://doi.org/10.1038/s41598-017-18893-8 |
| KX467296.1 | NG54 | India | https://doi.org/10.1038/s41598-017-18893-8 |
| KX467308.1 | NG30 | India | https://doi.org/10.1038/s41598-017-18893-8 |
| KX467309.1 | NG50 | India | https://doi.org/10.1038/s41598-017-18893-8 |
| KX467311.1 | NG197 | India | https://doi.org/10.1038/s41598-017-18893-8 |
| KX467300.1 | NG70 | India | https://doi.org/10.1038/s41598-017-18893-8 |
| KX467298.1 | NG32 | India | https://doi.org/10.1038/s41598-017-18893-8 |
| KX467295.1 | NG74 | India | https://doi.org/10.1038/s41598-017-18893-8 |
| KX467294.1 | NG40 | India | https://doi.org/10.1038/s41598-017-18893-8 |
| KX467297.1 | NG3 | India | https://doi.org/10.1038/s41598-017-18893-8 |
| KX467287.1 | NG118 | India | https://doi.org/10.1038/s41598-017-18893-8 |
| KX467284.1 | NG25 | India | https://doi.org/10.1038/s41598-017-18893-8 |
| KX467285.1 | NG21 | India | https://doi.org/10.1038/s41598-017-18893-8 |
| KX467280.1 | NG57 | India | https://doi.org/10.1038/s41598-017-18893-8 |
| KX467266.1 | NG49 | India | https://doi.org/10.1038/s41598-017-18893-8 |
| KX467267.1 | NG63 | India | https://doi.org/10.1038/s41598-017-18893-8 |
| KX467281.1 | NG1 | India | https://doi.org/10.1038/s41598-017-18893-8 |
| KX467288.1 | NG117 | India | https://doi.org/10.1038/s41598-017-18893-8 |
| KU178921.1 | NG115 | India | https://doi.org/10.1038/s41598-017-18893-8 |
| KU178917.1 | NG4 | India | https://doi.org/10.1038/s41598-017-18893-8 |
| KU178919.1 | NG47 | India | https://doi.org/10.1038/s41598-017-18893-8 |
| KU178922.1 | NG117 | India | https://doi.org/10.1038/s41598-017-18893-8 |
| KU178931.1 | NG119 | India | https://doi.org/10.1038/s41598-017-18893-8 |
| KU178928.1 | NG45 | India | https://doi.org/10.1038/s41598-017-18893-8 |
| KU178926.1 | NG20 | India | https://doi.org/10.1038/s41598-017-18893-8 |
| KU178929.1 | NG84 | India | https://doi.org/10.1038/s41598-017-18893-8 |
| KX467262.1 | NG97 | India | https://doi.org/10.1038/s41598-017-18893-8 |
| KU178918.1 | NG29 | India | https://doi.org/10.1038/s41598-017-18893-8 |
| KX467278.1 | NG193 | India | https://doi.org/10.1038/s41598-017-18893-8 |
| KX467269.1 | NG120 | India | https://doi.org/10.1038/s41598-017-18893-8 |

**Altitude range: 1001-1500 m**

| Accession number | Haplogroup | Country | Reference |
| --- | --- | --- | --- |
| KC911354.1 | L2a1f2 | Iran | 10.1371/journal.pone.0080673 |
| KC911395.1 | L2a1f2 | Iran | 10.1371/journal.pone.0080673 |
| KC911559.1 | M42b1b | Iran | 10.1371/journal.pone.0080673 |
| KC911517.1 | G2a3 | Iran | 10.1371/journal.pone.0080673 |
| JF742201.1 | Nepal_159 | Nepal | https://doi.org/10.1038/jhg.2012.8 |
| JF742204.1 | Nepal_075 | Nepal | https://doi.org/10.1038/jhg.2012.8 |
| JF742203.1 | Nepal_152 | Nepal | https://doi.org/10.1038/jhg.2012.8 |
| JF742198.2 | Nepal_166 | Nepal | https://doi.org/10.1038/jhg.2012.8 |
| JF742200.2 | Nepal_190 | Nepal | https://doi.org/10.1038/jhg.2012.8 |
| KC911603.1 | D4j5b | Iran | 10.1371/journal.pone.0080673 |
| KC911586.1 | D4b2b1 | Iran | 10.1371/journal.pone.0080673 |
| KX467317.1 | NG66 | India | https://doi.org/10.1038/s41598-017-18893-8 |
| KC911596.1 | M5a2a4 | Iran | 10.1371/journal.pone.0080673 |
| KC911351.1 | M5a2a4 | Iran | 10.1371/journal.pone.0080673 |
| KC911312.1 | M5a2a4 | Iran | 10.1371/journal.pone.0080673 |
| KX467312.1 | NG41 | India | https://doi.org/10.1038/s41598-017-18893-8 |
| JF742206.1 | Nepal_057 | Nepal | https://doi.org/10.1038/jhg.2012.8 |
| JF742211.1 | Nepal_168 | Nepal | https://doi.org/10.1038/jhg.2012.8 |
| JF742207.1 | Nepal_076 | Nepal | https://doi.org/10.1038/jhg.2012.8 |
| JF742209.2 | Nepal_102 | Nepal | https://doi.org/10.1038/jhg.2012.8 |
| JF742208.2 | Nepal_198 | Nepal | https://doi.org/10.1038/jhg.2012.8 |
| JF742215.1 | Nepal_056 | Nepal | https://doi.org/10.1038/jhg.2012.8 |
| KC911475.1 | M18a | Iran | 10.1371/journal.pone.0080673 |
| JF742214.1 | Nepal_107 | Nepal | https://doi.org/10.1038/jhg.2012.8 |
| JF742213.1 | Nepal_045 | Nepal | https://doi.org/10.1038/jhg.2012.8 |
| JF742212.1 | Nepal_022 | Nepal | https://doi.org/10.1038/jhg.2012.8 |
| KX467291.1 | NG65 | India | https://doi.org/10.1038/s41598-017-18893-8 |
| KC911530.1 | M3a1 | Iran | 10.1371/journal.pone.0080673 |
| JF742210.1 | Nepal_079 | Nepal | https://doi.org/10.1038/jhg.2012.8 |
| JF742216.1 | Nepal_193 | Nepal | https://doi.org/10.1038/jhg.2012.8 |
| KC911567.1 | U2e2 | Iran | 10.1371/journal.pone.0080673 |
| KC911491.1 | U3a3 | Iran | 10.1371/journal.pone.0080673 |
| KC911305.1 | U3a2* | Iran | 10.1371/journal.pone.0080673 |
| KC911419.1 | W5 | Iran | 10.1371/journal.pone.0080673 |
| KC911592.1 | W1c | Iran | 10.1371/journal.pone.0080673 |
| KC911449.1 | W6 | Iran | 10.1371/journal.pone.0080673 |
| KC911370.1 | W6 | Iran | 10.1371/journal.pone.0080673 |
| KC911547.1 | W6 | Iran | 10.1371/journal.pone.0080673 |
| KC911604.1 | W6 | Iran | 10.1371/journal.pone.0080673 |
| KC911336.1 | F1b1* | Iran | 10.1371/journal.pone.0080673 |
| KC911393.1 | K2a5* | Iran | 10.1371/journal.pone.0080673 |
| KC911572.1 | K1b1c | Iran | 10.1371/journal.pone.0080673 |
| KC911628.1 | U1b2 | Iran | 10.1371/journal.pone.0080673 |
| KC911388.1 | U1a3a | Iran | 10.1371/journal.pone.0080673 |
| KC911306.1 | U1a3b1 | Iran | 10.1371/journal.pone.0080673 |
| KC911521.1 | J1b* | Iran | 10.1371/journal.pone.0080673 |
| KC911307.1 | J1b3ab1 | Iran | 10.1371/journal.pone.0080673 |
| KC911371.1 | J1c2e1 | Iran | 10.1371/journal.pone.0080673 |
| KC911436.1 | J1c7a | Iran | 10.1371/journal.pone.0080673 |
| JN857034.1 | Bt_124 | China | 10.1371/journal.pone.0032179 |
| KC911315.1 | U2c1 | Iran | 10.1371/journal.pone.0080673 |
| JF742197.1 | Nepal_028 | Nepal | https://doi.org/10.1038/jhg.2012.8 |
| KC911614.1 | I2* | Iran | 10.1371/journal.pone.0080673 |
| KC911414.1 | T2d1b | Iran | 10.1371/journal.pone.0080673 |
| KC911546.1 | T1a2* | Iran | 10.1371/journal.pone.0080673 |
| KC911350.1 | T1a1m | Iran | 10.1371/journal.pone.0080673 |
| KC911428.1 | T1a1m1 | Iran | 10.1371/journal.pone.0080673 |
| KC911501.1 | T2b28 | Iran | 10.1371/journal.pone.0080673 |
| KC911444.1 | T2a2b | Iran | 10.1371/journal.pone.0080673 |
| KC911608.1 | T2b31 | Iran | 10.1371/journal.pone.0080673 |
| KC911583.1 | T2b4'21* | Iran | 10.1371/journal.pone.0080673 |
| KC911460.1 | U4b1b1* | Iran | 10.1371/journal.pone.0080673 |
| KC911509.1 | U7a2a1 | Iran | 10.1371/journal.pone.0080673 |
| KC911347.1 | U7a4a1a | Iran | 10.1371/journal.pone.0080673 |
| KC911622.1 | U7a* | Iran | 10.1371/journal.pone.0080673 |
| KC911455.1 | U7a3a1 | Iran | 10.1371/journal.pone.0080673 |
| KC911526.1 | U7a* | Iran | 10.1371/journal.pone.0080673 |
| KC911287.1 | U7a* | Iran | 10.1371/journal.pone.0080673 |
| KC911488.1 | N1a3 | Iran | 10.1371/journal.pone.0080673 |
| KC911323.1 | N1a3 | Iran | 10.1371/journal.pone.0080673 |
| KC911478.1 | X2f | Iran | 10.1371/journal.pone.0080673 |
| JN857033.1 | Bt_122 | China | 10.1371/journal.pone.0032179 |
| KC911280.1 | R2a* | Iran | 10.1371/journal.pone.0080673 |
| KC911372.1 | HV2a2* | Iran | 10.1371/journal.pone.0080673 |
| JN857031.1 | Bt_94 | China | 10.1371/journal.pone.0032179 |
| KC911447.1 | R0a1a* | Iran | 10.1371/journal.pone.0080673 |
| KC911450.1 | HV1b3 | Iran | 10.1371/journal.pone.0080673 |
| KC911300.1 | H14b2 | Iran | 10.1371/journal.pone.0080673 |
| KC911443.1 | H13a2a2 | Iran | 10.1371/journal.pone.0080673 |
| KC911458.1 | H13a2a2 | Iran | 10.1371/journal.pone.0080673 |
| KC911425.1 | H5f | Iran | 10.1371/journal.pone.0080673 |
| KC911276.1 | H13c2 | Iran | 10.1371/journal.pone.0080673 |
| KC911612.1 | HV13b | Iran | 10.1371/journal.pone.0080673 |
| KC911298.1 | HV13b | Iran | 10.1371/journal.pone.0080673 |
| KC911606.1 | H3h2 | Iran | 10.1371/journal.pone.0080673 |
| KC911421.1 | HV9 | Iran | 10.1371/journal.pone.0080673 |
| KC911525.1 | HV* | Iran | 10.1371/journal.pone.0080673 |
| KC911472.1 | HV* | Iran | 10.1371/journal.pone.0080673 |
| JF742199.1 | Nepal_007 | Nepal | https://doi.org/10.1038/jhg.2012.8 |
| KC911430.1 | H20a | Iran | 10.1371/journal.pone.0080673 |
| KC911510.1 | H1ca | Iran | 10.1371/journal.pone.0080673 |
| KC911281.1 | H49b | Iran | 10.1371/journal.pone.0080673 |
| KC911467.1 | H* | Iran | 10.1371/journal.pone.0080673 |
| KC911500.1 | H3t | Iran | 10.1371/journal.pone.0080673 |
| KC911619.1 | H57 | Iran | 10.1371/journal.pone.0080673 |

**Altitude range: 1501-2000 m**

| Accession number | Haplogroup | Country | Reference |
| --- | --- | --- | --- |
| KC911426.1 | M2c | Iran | 10.1371/journal.pone.0080673 |
| KC911474.1 | H57 | Iran | 10.1371/journal.pone.0080673 |
| KC911304.1 | J1b1b1* | Iran | 10.1371/journal.pone.0080673 |
| KC911590.1 | J1b1b1* | Iran | 10.1371/journal.pone.0080673 |
| KC911616.1 | T1a1m1 | Iran | 10.1371/journal.pone.0080673 |
| KC911288.1 | U7a4a1a | Iran | 10.1371/journal.pone.0080673 |
| KC911587.1 | T1a1* | Iran | 10.1371/journal.pone.0080673 |
| KC911343.1 | T1a* | Iran | 10.1371/journal.pone.0080673 |
| KC911320.1 | T1a1* | Iran | 10.1371/journal.pone.0080673 |
| KC911342.1 | T2m | Iran | 10.1371/journal.pone.0080673 |
| KC911302.1 | T2a2b | Iran | 10.1371/journal.pone.0080673 |
| KC911397.1 | T2b31 | Iran | 10.1371/journal.pone.0080673 |
| KC911322.1 | X2* | Iran | 10.1371/journal.pone.0080673 |
| KC911480.1 | X2e2c | Iran | 10.1371/journal.pone.0080673 |
| KC911316.1 | J1d6* | Iran | 10.1371/journal.pone.0080673 |
| KC911461.1 | J1b3b1 | Iran | 10.1371/journal.pone.0080673 |
| KC911401.1 | J1b6* | Iran | 10.1371/journal.pone.0080673 |
| KC911544.1 | J1b5a | Iran | 10.1371/journal.pone.0080673 |
| KC911381.1 | J1b4a1 | Iran | 10.1371/journal.pone.0080673 |
| KC911407.1 | J1b3* | Iran | 10.1371/journal.pone.0080673 |
| KC911366.1 | J1b6a | Iran | 10.1371/journal.pone.0080673 |
| KC911431.1 | N2a1 | Iran | 10.1371/journal.pone.0080673 |
| KC911433.1 | W1c | Iran | 10.1371/journal.pone.0080673 |
| KC911363.1 | M5a2a1a | Iran | 10.1371/journal.pone.0080673 |
| KC911585.1 | U4b* | Iran | 10.1371/journal.pone.0080673 |
| KC911489.1 | U2d2 | Iran | 10.1371/journal.pone.0080673 |
| KC911493.1 | HV2a1 | Iran | 10.1371/journal.pone.0080673 |
| KC911331.1 | HV2a1 | Iran | 10.1371/journal.pone.0080673 |
| KC911514.1 | HV2a1 | Iran | 10.1371/journal.pone.0080673 |
| KX467279.1 | NG211 | India | https://doi.org/10.1038/s41598-017-18893-8 |
| KC911582.1 | U5a1a1* | Iran | 10.1371/journal.pone.0080673 |
| KC911374.1 | R2a* | Iran | 10.1371/journal.pone.0080673 |
| KC911495.1 | R2a4 | Iran | 10.1371/journal.pone.0080673 |
| KC911337.1 | R2a4 | Iran | 10.1371/journal.pone.0080673 |
| KC911485.1 | R2a4 | Iran | 10.1371/journal.pone.0080673 |
| KC911373.1 | R0a2* | Iran | 10.1371/journal.pone.0080673 |
| KX467272.1 | NG207 | India | https://doi.org/10.1038/s41598-017-18893-8 |
| KX467270.1 | NG134 | India | https://doi.org/10.1038/s41598-017-18893-8 |
| KX467268.1 | NG104 | India | https://doi.org/10.1038/s41598-017-18893-8 |
| KC911391.1 | HV* | Iran | 10.1371/journal.pone.0080673 |
| KC911597.1 | HV1a3 | Iran | 10.1371/journal.pone.0080673 |
| KC911338.1 | H13a2c* | Iran | 10.1371/journal.pone.0080673 |
| KC911294.1 | H63* | Iran | 10.1371/journal.pone.0080673 |
| KC911303.1 | H* | Iran | 10.1371/journal.pone.0080673 |
| KC911469.1 | H13a2b4 | Iran | 10.1371/journal.pone.0080673 |
| KC911286.1 | H10* | Iran | 10.1371/journal.pone.0080673 |
| KC911403.1 | H7b7 | Iran | 10.1371/journal.pone.0080673 |
| KC911560.1 | HV* | Iran | 10.1371/journal.pone.0080673 |
| KC911340.1 | HV18 | Iran | 10.1371/journal.pone.0080673 |
| KC911471.1 | HV18 | Iran | 10.1371/journal.pone.0080673 |
| KC911277.1 | H1ca | Iran | 10.1371/journal.pone.0080673 |
| KC911318.1 | H1ca | Iran | 10.1371/journal.pone.0080673 |
| KC911565.1 | H1* | Iran | 10.1371/journal.pone.0080673 |
| KC911352.1 | H* | Iran | 10.1371/journal.pone.0080673 |
| KC911591.1 | H* | Iran | 10.1371/journal.pone.0080673 |
| KC911406.1 | V* | Iran | 10.1371/journal.pone.0080673 |
| KC911580.1 | H* | Iran | 10.1371/journal.pone.0080673 |
| KC911548.1 | H14b* | Iran | 10.1371/journal.pone.0080673 |
| KC911617.1 | H2a1h | Iran | 10.1371/journal.pone.0080673 |
| KC911598.1 | H2a1h | Iran | 10.1371/journal.pone.0080673 |
| KC911386.1 | H2a1* | Iran | 10.1371/journal.pone.0080673 |
| KC911364.1 | L5c1 | Iran | 10.1371/journal.pone.0080673 |
| KC911377.1 | T2c1a* | Iran | 10.1371/journal.pone.0080673 |
| KC911490.1 | T2b* | Iran | 10.1371/journal.pone.0080673 |
| KC911362.1 | T2i1 | Iran | 10.1371/journal.pone.0080673 |
| KC911481.1 | T2i2 | Iran | 10.1371/journal.pone.0080673 |
| KC911507.1 | T2i2 | Iran | 10.1371/journal.pone.0080673 |
| KC911357.1 | T2i2 | Iran | 10.1371/journal.pone.0080673 |
| KC911499.1 | T2i2 | Iran | 10.1371/journal.pone.0080673 |
| KC911314.1 | U3a2a1 | Iran | 10.1371/journal.pone.0080673 |
| KC911365.1 | U1a3a | Iran | 10.1371/journal.pone.0080673 |
| KC911437.1 | U1a1a1 | Iran | 10.1371/journal.pone.0080673 |
| KC911344.1 | U1a1a | Iran | 10.1371/journal.pone.0080673 |
| KC911602.1 | U2d1 | Iran | 10.1371/journal.pone.0080673 |
| KU178925.1 | NG212 | India | https://doi.org/10.1038/s41598-017-18893-8 |
| KC911610.1 | J1b1a3 | Iran | 10.1371/journal.pone.0080673 |
| KC911625.1 | J1b1b1* | Iran | 10.1371/journal.pone.0080673 |
| KC911599.1 | X2e2c | Iran | 10.1371/journal.pone.0080673 |
| KC911375.1 | U4c1a | Iran | 10.1371/journal.pone.0080673 |
| KC911396.1 | U4c1a | Iran | 10.1371/journal.pone.0080673 |
| KC911492.1 | N3* | Iran | 10.1371/journal.pone.0080673 |
| KC911348.1 | N1b1a | Iran | 10.1371/journal.pone.0080673 |
| KX467276.1 | NG34 | India | https://doi.org/10.1038/s41598-017-18893-8 |
| KC911551.1 | R5a2b* | Iran | 10.1371/journal.pone.0080673 |
| KC911284.1 | H5a1* | Iran | 10.1371/journal.pone.0080673 |
| KU178930.1 | NG111 | India | https://doi.org/10.1038/s41598-017-18893-8 |
| KC911563.1 | U7a3a1 | Iran | 10.1371/journal.pone.0080673 |
| KC911620.1 | U7a1* | Iran | 10.1371/journal.pone.0080673 |
| AY255178.2 | YN163 | China | 10.1086/377718 |
| KC911629.1 | C5c1b | Iran | 10.1371/journal.pone.0080673 |
| KC911435.1 | I1a* | Iran | 10.1371/journal.pone.0080673 |
| KX467275.1 | NG206 | India | https://doi.org/10.1038/s41598-017-18893-8 |
| KC911321.1 | M5a2a4 | Iran | 10.1371/journal.pone.0080673 |
| KC911557.1 | M5a2a4 | Iran | 10.1371/journal.pone.0080673 |
| KX467313.1 | NG107 | India | https://doi.org/10.1038/s41598-017-18893-8 |
| KX467314.1 | NG79 | India | https://doi.org/10.1038/s41598-017-18893-8 |
| KX467302.1 | NG98 | India | https://doi.org/10.1038/s41598-017-18893-8 |
| KX467305.1 | NG31 | India | https://doi.org/10.1038/s41598-017-18893-8 |
| KX467292.1 | NG105 | India | https://doi.org/10.1038/s41598-017-18893-8 |
| KX467293.1 | NG103 | India | https://doi.org/10.1038/s41598-017-18893-8 |
| KX467310.1 | NG100 | India | https://doi.org/10.1038/s41598-017-18893-8 |
| KX467299.1 | NG56 | India | https://doi.org/10.1038/s41598-017-18893-8 |
| KX467301.1 | NG71 | India | https://doi.org/10.1038/s41598-017-18893-8 |

**Altitude range: 2001-2500 m**

| Accession number | Haplogroup | Country | Reference |
| --- | --- | --- | --- |
| KF056257.1 | XEB151 | China | 10.1093/molbev/mst147 |
| KU178920.1 | NG64 | India | https://doi.org/10.1038/s41598-017-18893-8 |
| KF056260.1 | XEB152 | China | 10.1093/molbev/mst147 |
| KF056259.1 | XEB108 | China | 10.1093/molbev/mst147 |
| KF056258.1 | XEB072 | China | 10.1093/molbev/mst147 |
| KF056261.1 | XEB124 | China | 10.1093/molbev/mst147 |
| KF056262.1 | XEB035 | China | 10.1093/molbev/mst147 |
| KF056299.1 | XEB111 | China | 10.1093/molbev/mst147 |
| KF056298.1 | XEB009 | China | 10.1093/molbev/mst147 |
| KF056301.1 | XEB115 | China | 10.1093/molbev/mst147 |
| KF056300.1 | XEB024 | China | 10.1093/molbev/mst147 |
| KF056303.1 | XEB161 | China | 10.1093/molbev/mst147 |
| KF056302.1 | XEB160 | China | 10.1093/molbev/mst147 |
| KF056305.1 | XEB023 | China | 10.1093/molbev/mst147 |
| KF056306.1 | XEB059 | China | 10.1093/molbev/mst147 |
| KF056307.1 | XEB184 | China | 10.1093/molbev/mst147 |
| KF056308.1 | XEB062 | China | 10.1093/molbev/mst147 |
| KF056309.1 | XEB013 | China | 10.1093/molbev/mst147 |
| KF056317.1 | XEB131 | China | 10.1093/molbev/mst147 |
| KF056316.1 | XEB077 | China | 10.1093/molbev/mst147 |
| KF056315.1 | XEB075 | China | 10.1093/molbev/mst147 |
| KF056314.1 | XEB073 | China | 10.1093/molbev/mst147 |
| KF056313.1 | XEB056 | China | 10.1093/molbev/mst147 |
| KF056312.1 | XEB125 | China | 10.1093/molbev/mst147 |
| KF056311.1 | XEB114 | China | 10.1093/molbev/mst147 |
| KF056310.1 | XEB004 | China | 10.1093/molbev/mst147 |
| KF056304.1 | XEB150 | China | 10.1093/molbev/mst147 |
| KF056246.1 | XEB008 | China | 10.1093/molbev/mst147 |
| KF056247.1 | XEB102 | China | 10.1093/molbev/mst147 |
| KF056256.1 | XEB164 | China | 10.1093/molbev/mst147 |
| KF056255.1 | XEB174 | China | 10.1093/molbev/mst147 |
| KF056254.1 | XEB166 | China | 10.1093/molbev/mst147 |
| KF056253.1 | XEB162 | China | 10.1093/molbev/mst147 |
| KF056252.1 | XEB145 | China | 10.1093/molbev/mst147 |
| KF056251.1 | XEB043 | China | 10.1093/molbev/mst147 |
| KF056250.1 | XEB017 | China | 10.1093/molbev/mst147 |
| KF056249.1 | XEB014 | China | 10.1093/molbev/mst147 |
| KF056248.1 | XEB003 | China | 10.1093/molbev/mst147 |
| KF056245.1 | XEB130 | China | 10.1093/molbev/mst147 |
| KF056244.1 | XEB126 | China | 10.1093/molbev/mst147 |
| KF056243.1 | XEB057 | China | 10.1093/molbev/mst147 |
| KC911353.1 | K1a* | Iran | 10.1371/journal.pone.0080673 |
| KC911465.1 | X2e2c | Iran | 10.1371/journal.pone.0080673 |
| KC911497.1 | X2* | Iran | 10.1371/journal.pone.0080673 |
| KC911317.1 | N1a3 | Iran | 10.1371/journal.pone.0080673 |
| KC911368.1 | N2a2 | Iran | 10.1371/journal.pone.0080673 |
| KC911627.1 | R2a4 | Iran | 10.1371/journal.pone.0080673 |
| KF056318.1 | XEB129 | China | 10.1093/molbev/mst147 |
| KC911456.1 | HV14 | Iran | 10.1371/journal.pone.0080673 |
| KC911297.1 | H1ca | Iran | 10.1371/journal.pone.0080673 |
| KC911283.1 | H1ca | Iran | 10.1371/journal.pone.0080673 |
| KC911413.1 | H* | Iran | 10.1371/journal.pone.0080673 |
| KF056264.1 | XEB180 | China | 10.1093/molbev/mst147 |
| KF056263.1 | XEB005 | China | 10.1093/molbev/mst147 |
| KC911394.1 | M4b | Iran | 10.1371/journal.pone.0080673 |
| KC911579.1 | M5a2a4 | Iran | 10.1371/journal.pone.0080673 |
| KF056268.1 | XEB031 | China | 10.1093/molbev/mst147 |
| KF056267.1 | XEB047 | China | 10.1093/molbev/mst147 |
| KF056266.1 | XEB002 | China | 10.1093/molbev/mst147 |
| KF056269.1 | XEB029 | China | 10.1093/molbev/mst147 |
| KF056274.1 | XEB101 | China | 10.1093/molbev/mst147 |
| KF056265.1 | XEB051 | China | 10.1093/molbev/mst147 |
| KF056272.1 | XEB042 | China | 10.1093/molbev/mst147 |
| KF056271.1 | XEB012 | China | 10.1093/molbev/mst147 |
| KF056275.1 | XEB110 | China | 10.1093/molbev/mst147 |
| KF056279.1 | XEB049 | China | 10.1093/molbev/mst147 |
| KF056278.1 | XEB149 | China | 10.1093/molbev/mst147 |
| KF056277.1 | XEB076 | China | 10.1093/molbev/mst147 |
| KF056276.1 | XEB050 | China | 10.1093/molbev/mst147 |
| KF056270.1 | XEB006 | China | 10.1093/molbev/mst147 |
| KF056273.1 | XEB048 | China | 10.1093/molbev/mst147 |
| KF056281.1 | XEB139 | China | 10.1093/molbev/mst147 |
| KF056283.1 | XEB103 | China | 10.1093/molbev/mst147 |
| KF056282.1 | XEB026 | China | 10.1093/molbev/mst147 |
| KF056280.1 | XEB036 | China | 10.1093/molbev/mst147 |
| KF056286.1 | XEB104 | China | 10.1093/molbev/mst147 |
| KF056285.1 | XEB039 | China | 10.1093/molbev/mst147 |
| KF056284.1 | XEB020 | China | 10.1093/molbev/mst147 |
| KF056292.1 | XEB185 | China | 10.1093/molbev/mst147 |
| KF056291.1 | XEB058 | China | 10.1093/molbev/mst147 |
| KF056287.1 | XEB116 | China | 10.1093/molbev/mst147 |
| KF056288.1 | XEB015 | China | 10.1093/molbev/mst147 |
| KF056289.1 | XEB171 | China | 10.1093/molbev/mst147 |
| KF056290.1 | XEB163 | China | 10.1093/molbev/mst147 |
| KF056293.1 | XEB100 | China | 10.1093/molbev/mst147 |
| KF056294.1 | XEB170 | China | 10.1093/molbev/mst147 |
| KF056297.1 | XEB158 | China | 10.1093/molbev/mst147 |
| KF056296.1 | XEB113 | China | 10.1093/molbev/mst147 |
| KF056295.1 | XEB098 | China | 10.1093/molbev/mst147 |

**Altitude range: 2501-3000 m**

| Accession number | Haplogroup | Country | Reference |
| --- | --- | --- | --- |
| KT725933.1 | DB072 | Zayu County, Nyingrtri Prefecture | 10.1038/srep31083 |
| KT725923.1 | DB062 | Zayu County, Nyingrtri Prefecture | 10.1038/srep31083 |
| KT725940.1 | DB080 | Zayu County, Nyingrtri Prefecture | 10.1038/srep31083 |
| KT725898.1 | DB036 | Zayu County, Nyingrtri Prefecture | 10.1038/srep31083 |
| KT725906.1 | DB045 | Zayu County, Nyingrtri Prefecture | 10.1038/srep31083 |
| KT725902.1 | DB040 | Zayu County, Nyingrtri Prefecture | 10.1038/srep31083 |
| KT725944.1 | DB086 | Zayu County, Nyingrtri Prefecture | 10.1038/srep31083 |
| KT725885.1 | DB020 | Zayu County, Nyingrtri Prefecture | 10.1038/srep31083 |
| KT725929.1 | DB068 | Zayu County, Nyingrtri Prefecture | 10.1038/srep31083 |
| KT725925.1 | DB064 | Zayu County, Nyingrtri Prefecture | 10.1038/srep31083 |
| KT725883.1 | DB018 | Zayu County, Nyingrtri Prefecture | 10.1038/srep31083 |
| KT725920.1 | DB059 | Zayu County, Nyingrtri Prefecture | 10.1038/srep31083 |
| KT725954.1 | DB098 | Zayu County, Nyingrtri Prefecture | 10.1038/srep31083 |
| KT725952.1 | DB096 | Zayu County, Nyingrtri Prefecture | 10.1038/srep31083 |
| KT725922.1 | DB061 | Zayu County, Nyingrtri Prefecture | 10.1038/srep31083 |
| KT725905.1 | DB044 | Zayu County, Nyingrtri Prefecture | 10.1038/srep31083 |
| KT725876.1 | DB011 | Zayu County, Nyingrtri Prefecture | 10.1038/srep31083 |
| KT725949.1 | DB093 | Zayu County, Nyingrtri Prefecture | 10.1038/srep31083 |
| KT725888.1 | DB023 | Zayu County, Nyingrtri Prefecture | 10.1038/srep31083 |
| KT725948.1 | DB092 | Zayu County, Nyingrtri Prefecture | 10.1038/srep31083 |
| KT725932.1 | DB071 | Zayu County, Nyingrtri Prefecture | 10.1038/srep31083 |
| KT725896.1 | DB034 | Zayu County, Nyingrtri Prefecture | 10.1038/srep31083 |
| KT725889.1 | DB024 | Zayu County, Nyingrtri Prefecture | 10.1038/srep31083 |
| KT725938.1 | DB077 | Zayu County, Nyingrtri Prefecture | 10.1038/srep31083 |
| KX467283.1 | NG145 | India | 10.1038/s41598-017-18893-8 |
| KT725880.1 | DB015 | Zayu County, Nyingrtri Prefecture | 10.1038/srep31083 |
| KT725899.1 | DB037 | Zayu County, Nyingrtri Prefecture | 10.1038/srep31083 |
| KT725941.1 | DB081 | Zayu County, Nyingrtri Prefecture | 10.1038/srep31083 |
| KT725937.1 | DB076 | Zayu County, Nyingrtri Prefecture | 10.1038/srep31083 |
| KT725914.1 | DB053 | Zayu County, Nyingrtri Prefecture | 10.1038/srep31083 |
| KT725921.1 | DB060 | Zayu County, Nyingrtri Prefecture | 10.1038/srep31083 |
| KT725936.1 | DB075 | Zayu County, Nyingrtri Prefecture | 10.1038/srep31083 |
| KT725951.1 | DB095 | Zayu County, Nyingrtri Prefecture | 10.1038/srep31083 |
| KT725924.1 | DB063 | Zayu County, Nyingrtri Prefecture | 10.1038/srep31083 |
| KT725935.1 | DB074 | Zayu County, Nyingrtri Prefecture | 10.1038/srep31083 |
| KT725908.1 | DB047 | Zayu County, Nyingrtri Prefecture | 10.1038/srep31083 |
| KT725891.1 | DB027 | Zayu County, Nyingrtri Prefecture | 10.1038/srep31083 |
| KT725901.1 | DB039 | Zayu County, Nyingrtri Prefecture | 10.1038/srep31083 |
| KT725884.1 | DB019 | Zayu County, Nyingrtri Prefecture | 10.1038/srep31083 |
| KT725895.1 | DB033 | Zayu County, Nyingrtri Prefecture | 10.1038/srep31083 |
| KT725879.1 | DB014 | Zayu County, Nyingrtri Prefecture | 10.1038/srep31083 |
| KT725878.1 | DB013 | Zayu County, Nyingrtri Prefecture | 10.1038/srep31083 |
| KT725931.1 | DB070 | Zayu County, Nyingrtri Prefecture | 10.1038/srep31083 |
| KT725881.1 | DB016 | Zayu County, Nyingrtri Prefecture | 10.1038/srep31083 |
| KT725917.1 | DB056 | Zayu County, Nyingrtri Prefecture | 10.1038/srep31083 |
| KT725897.1 | DB035 | Zayu County, Nyingrtri Prefecture | 10.1038/srep31083 |
| KT725892.1 | DB030 | Zayu County, Nyingrtri Prefecture | 10.1038/srep31083 |
| KT725887.1 | DB022 | Zayu County, Nyingrtri Prefecture | 10.1038/srep31083 |
| KT725912.1 | DB051 | Zayu County, Nyingrtri Prefecture | 10.1038/srep31083 |
| KT725875.1 | DB010 | Zayu County, Nyingrtri Prefecture | 10.1038/srep31083 |
| KT725894.1 | DB032 | Zayu County, Nyingrtri Prefecture | 10.1038/srep31083 |
| KT725946.1 | DB088 | Zayu County, Nyingrtri Prefecture | 10.1038/srep31083 |
| KT725947.1 | DB089 | Zayu County, Nyingrtri Prefecture | 10.1038/srep31083 |
| KT725957.1 | DB101 | Zayu County, Nyingrtri Prefecture | 10.1038/srep31083 |
| KT725913.1 | DB052 | Zayu County, Nyingrtri Prefecture | 10.1038/srep31083 |
| KT725915.1 | DB054 | Zayu County, Nyingrtri Prefecture | 10.1038/srep31083 |
| KT725945.1 | DB087 | Zayu County, Nyingrtri Prefecture | 10.1038/srep31083 |
| KT725943.1 | DB084 | Zayu County, Nyingrtri Prefecture | 10.1038/srep31083 |
| KT725900.1 | DB038 | Zayu County, Nyingrtri Prefecture | 10.1038/srep31083 |
| KT725953.1 | DB097 | Zayu County, Nyingrtri Prefecture | 10.1038/srep31083 |
| KT725958.1 | DB102 | Zayu County, Nyingrtri Prefecture | 10.1038/srep31083 |
| KT725904.1 | DB043 | Zayu County, Nyingrtri Prefecture | 10.1038/srep31083 |
| KT725886.1 | DB021 | Zayu County, Nyingrtri Prefecture | 10.1038/srep31083 |
| KT725874.1 | DB009 | Zayu County, Nyingrtri Prefecture | 10.1038/srep31083 |
| KT725872.1 | DB007 | Zayu County, Nyingrtri Prefecture | 10.1038/srep31083 |
| KT725955.1 | DB099 | Zayu County, Nyingrtri Prefecture | 10.1038/srep31083 |
| KT725928.1 | DB067 | Zayu County, Nyingrtri Prefecture | 10.1038/srep31083 |
| KT725869.1 | DB003 | Zayu County, Nyingrtri Prefecture | 10.1038/srep31083 |
| KT725939.1 | DB079 | Zayu County, Nyingrtri Prefecture | 10.1038/srep31083 |
| KT725871.1 | DB006 | Zayu County, Nyingrtri Prefecture | 10.1038/srep31083 |
| KX467264.1 | NG139 | India | 10.1038/s41598-017-18893-8 |
| KX467271.1 | NG136 | India | 10.1038/s41598-017-18893-8 |
| KX467324.1 | NG140 | India | 10.1038/s41598-017-18893-8 |
| KX467327.1 | NG143 | India | 10.1038/s41598-017-18893-8 |
| KT725918.1 | DB057 | Zayu County, Nyingrtri Prefecture | 10.1038/srep31083 |
| KT725907.1 | DB046 | Zayu County, Nyingrtri Prefecture | 10.1038/srep31083 |
| KT725873.1 | DB008 | Zayu County, Nyingrtri Prefecture | 10.1038/srep31083 |
| KT725930.1 | DB069 | Zayu County, Nyingrtri Prefecture | 10.1038/srep31083 |
| KT725909.1 | DB048 | Zayu County, Nyingrtri Prefecture | 10.1038/srep31083 |
| KT725956.1 | DB100 | Zayu County, Nyingrtri Prefecture | 10.1038/srep31083 |
| KT725882.1 | DB017 | Zayu County, Nyingrtri Prefecture | 10.1038/srep31083 |
| KT725890.1 | DB025 | Zayu County, Nyingrtri Prefecture | 10.1038/srep31083 |
| KT725893.1 | DB031 | Zayu County, Nyingrtri Prefecture | 10.1038/srep31083 |
| KT725934.1 | DB073 | Zayu County, Nyingrtri Prefecture | 10.1038/srep31083 |
| KT725926.1 | DB065 | Zayu County, Nyingrtri Prefecture | 10.1038/srep31083 |
| KT725868.1 | DB001 | Zayu County, Nyingrtri Prefecture | 10.1038/srep31083 |

**Altitude range: 3001-4000 m**

| Accession number | Haplogroup | Country | Reference |
| --- | --- | --- | --- |
| KT726145.1 | MB597 | Nyingrtri Prefecture | 10.1038/srep31083 |
| KT726096.1 | LB190 | Nyingrtri Prefecture | 10.1038/srep31083 |
| KT726126.1 | LB331 | Nyingrtri Prefecture | 10.1038/srep31083 |
| KT726138.1 | MB570 | Nyingrtri Prefecture | 10.1038/srep31083 |
| KT726104.1 | LB200 | Nyingrtri Prefecture | 10.1038/srep31083 |
| KT726051.1 | LB115 | Nyingrtri Prefecture | 10.1038/srep31083 |
| KT726139.1 | MB571 | Nyingrtri Prefecture | 10.1038/srep31083 |
| KT726131.1 | LB577 | Nyingrtri Prefecture | 10.1038/srep31083 |
| KT726128.1 | LB345 | Nyingrtri Prefecture | 10.1038/srep31083 |
| KT726127.1 | LB344 | Nyingrtri Prefecture | 10.1038/srep31083 |
| KT726105.1 | LB202 | Nyingrtri Prefecture | 10.1038/srep31083 |
| KT726071.1 | LB155 | Nyingrtri Prefecture | 10.1038/srep31083 |
| KT726058.1 | LB124 | Nyingrtri Prefecture | 10.1038/srep31083 |
| KT726130.1 | LB348 | Nyingrtri Prefecture | 10.1038/srep31083 |
| KT726120.1 | LB260 | Nyingrtri Prefecture | 10.1038/srep31083 |
| KT726098.1 | LB192 | Nyingrtri Prefecture | 10.1038/srep31083 |
| KT726090.1 | LB184 | Nyingrtri Prefecture | 10.1038/srep31083 |
| KT726074.1 | LB160 | Nyingrtri Prefecture | 10.1038/srep31083 |
| KT726073.1 | LB159 | Nyingrtri Prefecture | 10.1038/srep31083 |
| KT726059.1 | LB125 | Nyingrtri Prefecture | 10.1038/srep31083 |
| KT726063.1 | LB138 | Nyingrtri Prefecture | 10.1038/srep31083 |
| KT726110.1 | LB208 | Nyingrtri Prefecture | 10.1038/srep31083 |
| KT726069.1 | LB147 | Nyingrtri Prefecture | 10.1038/srep31083 |
| KT726049.1 | LB113 | Nyingrtri Prefecture | 10.1038/srep31083 |
| KT726124.1 | LB324 | Nyingrtri Prefecture | 10.1038/srep31083 |
| KT726116.1 | LB249 | Nyingrtri Prefecture | 10.1038/srep31083 |
| KT726089.1 | LB183 | Nyingrtri Prefecture | 10.1038/srep31083 |
| KT726082.1 | LB170 | Nyingrtri Prefecture | 10.1038/srep31083 |
| KT726078.1 | LB166 | Nyingrtri Prefecture | 10.1038/srep31083 |
| KT726062.1 | LB137 | Nyingrtri Prefecture | 10.1038/srep31083 |
| KT726060.1 | LB127 | Nyingrtri Prefecture | 10.1038/srep31083 |
| KT726053.1 | LB118 | Nyingrtri Prefecture | 10.1038/srep31083 |
| KT726048.1 | LB112 | Nyingrtri Prefecture | 10.1038/srep31083 |
| KT726108.1 | LB206 | Nyingrtri Prefecture | 10.1038/srep31083 |
| KT726057.1 | LB122 | Nyingrtri Prefecture | 10.1038/srep31083 |
| KT726052.1 | LB117 | Nyingrtri Prefecture | 10.1038/srep31083 |
| KT726129.1 | LB346 | Nyingrtri Prefecture | 10.1038/srep31083 |
| KT726118.1 | LB251 | Nyingrtri Prefecture | 10.1038/srep31083 |
| KT726093.1 | LB187 | Nyingrtri Prefecture | 10.1038/srep31083 |
| KT726065.1 | LB140 | Nyingrtri Prefecture | 10.1038/srep31083 |
| KT726075.1 | LB162 | Nyingrtri Prefecture | 10.1038/srep31083 |
| KT726061.1 | LB136 | Nyingrtri Prefecture | 10.1038/srep31083 |
| KT726123.1 | LB288 | Nyingrtri Prefecture | 10.1038/srep31083 |
| KT726122.1 | LB285 | Nyingrtri Prefecture | 10.1038/srep31083 |
| KT726142.1 | MB593 | Nyingrtri Prefecture | 10.1038/srep31083 |
| KT726135.1 | LB611 | Nyingrtri Prefecture | 10.1038/srep31083 |
| KT726134.1 | LB586 | Nyingrtri Prefecture | 10.1038/srep31083 |
| KT726133.1 | LB585 | Nyingrtri Prefecture | 10.1038/srep31083 |
| KT726132.1 | LB582 | Nyingrtri Prefecture | 10.1038/srep31083 |
| KT726147.1 | MB600 | Nyingrtri Prefecture | 10.1038/srep31083 |
| KT726072.1 | LB156 | Nyingrtri Prefecture | 10.1038/srep31083 |
| KT726109.1 | LB207 | Nyingrtri Prefecture | 10.1038/srep31083 |
| KT726125.1 | LB327 | Nyingrtri Prefecture | 10.1038/srep31083 |
| KT726146.1 | MB598 | Nyingrtri Prefecture | 10.1038/srep31083 |
| KT726081.1 | LB169 | Nyingrtri Prefecture | 10.1038/srep31083 |
| KT726068.1 | LB143 | Nyingrtri Prefecture | 10.1038/srep31083 |
| KT726100.1 | LB195 | Nyingrtri Prefecture | 10.1038/srep31083 |
| KT726092.1 | LB186 | Nyingrtri Prefecture | 10.1038/srep31083 |
| KT726087.1 | LB180 | Nyingrtri Prefecture | 10.1038/srep31083 |
| KT726099.1 | LB193 | Nyingrtri Prefecture | 10.1038/srep31083 |
| KT726086.1 | LB177 | Nyingrtri Prefecture | 10.1038/srep31083 |
| KT726080.1 | LB168 | Nyingrtri Prefecture | 10.1038/srep31083 |
| KT726103.1 | LB199 | Nyingrtri Prefecture | 10.1038/srep31083 |
| KT726077.1 | LB164 | Nyingrtri Prefecture | 10.1038/srep31083 |
| KT726136.1 | MB298 | Nyingrtri Prefecture | 10.1038/srep31083 |
| KT726056.1 | LB121 | Nyingrtri Prefecture | 10.1038/srep31083 |
| KT726045.1 | LB109 | Nyingrtri Prefecture | 10.1038/srep31083 |
| KT726152.1 | MB609 | Nyingrtri Prefecture | 10.1038/srep31083 |
| KT726097.1 | LB191 | Nyingrtri Prefecture | 10.1038/srep31083 |
| KT726148.1 | MB601 | Nyingrtri Prefecture | 10.1038/srep31083 |
| KT726150.1 | MB606 | Nyingrtri Prefecture | 10.1038/srep31083 |
| KT726067.1 | LB142 | Nyingrtri Prefecture | 10.1038/srep31083 |
| KT726115.1 | LB244 | Nyingrtri Prefecture | 10.1038/srep31083 |
| KT726119.1 | LB258 | Nyingrtri Prefecture | 10.1038/srep31083 |
| KT726106.1 | LB203 | Nyingrtri Prefecture | 10.1038/srep31083 |
| KT726094.1 | LB188 | Nyingrtri Prefecture | 10.1038/srep31083 |
| KT726066.1 | LB141 | Nyingrtri Prefecture | 10.1038/srep31083 |
| KT726083.1 | LB171 | Nyingrtri Prefecture | 10.1038/srep31083 |
| KT726113.1 | LB228 | Nyingrtri Prefecture | 10.1038/srep31083 |
| KT726102.1 | LB198 | Nyingrtri Prefecture | 10.1038/srep31083 |
| KT726101.1 | LB197 | Nyingrtri Prefecture | 10.1038/srep31083 |
| KT726144.1 | MB595 | Nyingrtri Prefecture | 10.1038/srep31083 |
| KT726117.1 | LB250 | Nyingrtri Prefecture | 10.1038/srep31083 |
| KT726121.1 | LB282 | Nyingrtri Prefecture | 10.1038/srep31083 |
| KT726107.1 | LB204 | Nyingrtri Prefecture | 10.1038/srep31083 |
| KT726079.1 | LB167 | Nyingrtri Prefecture | 10.1038/srep31083 |
| KT726076.1 | LB163 | Nyingrtri Prefecture | 10.1038/srep31083 |
| KT726111.1 | LB221 | Nyingrtri Prefecture | 10.1038/srep31083 |
| KT726085.1 | LB176 | Nyingrtri Prefecture | 10.1038/srep31083 |
| KT726055.1 | LB120 | Nyingrtri Prefecture | 10.1038/srep31083 |
| KT726140.1 | MB583 | Nyingrtri Prefecture | 10.1038/srep31083 |
| KT726151.1 | MB608 | Nyingrtri Prefecture | 10.1038/srep31083 |
| KT726047.1 | LB111 | Nyingrtri Prefecture | 10.1038/srep31083 |
| KT726095.1 | LB189 | Nyingrtri Prefecture | 10.1038/srep31083 |
| KT726088.1 | LB181 | Nyingrtri Prefecture | 10.1038/srep31083 |
| KT726070.1 | LB154 | Nyingrtri Prefecture | 10.1038/srep31083 |
| KT726091.1 | LB185 | Nyingrtri Prefecture | 10.1038/srep31083 |
| KT726046.1 | LB110 | Nyingrtri Prefecture | 10.1038/srep31083 |
| KT726143.1 | MB594 | Nyingrtri Prefecture | 10.1038/srep31083 |
| KT726050.1 | LB114 | Nyingrtri Prefecture | 10.1038/srep31083 |
| KT726064.1 | LB139 | Nyingrtri Prefecture | 10.1038/srep31083 |
| KT726149.1 | MB602 | Nyingrtri Prefecture | 10.1038/srep31083 |
| KT726141.1 | MB592 | Nyingrtri Prefecture | 10.1038/srep31083 |
| KT726137.1 | MB328 | Nyingrtri Prefecture | 10.1038/srep31083 |
| KT726054.1 | LB119 | Nyingrtri Prefecture | 10.1038/srep31083 |
| KT726114.1 | LB232 | Nyingrtri Prefecture | 10.1038/srep31083 |
| KT726084.1 | LB175 | Nyingrtri Prefecture | 10.1038/srep31083 |

**Altitude range: >4000 m**

| Accession number | Haplogroup | Country | Reference |
| --- | --- | --- | --- |
| KT725993.1 | DR038 | Shigatse Prefecture | 10.1038/srep31083 |
| KT726007.1 | DR052 | Shigatse Prefecture | 10.1038/srep31083 |
| KT726037.1 | DR100 | Shigatse Prefecture | 10.1038/srep31083 |
| KT725989.1 | DR033 | Shigatse Prefecture | 10.1038/srep31083 |
| KT725966.1 | DR009 | Shigatse Prefecture | 10.1038/srep31083 |
| KT725975.1 | DR019 | Shigatse Prefecture | 10.1038/srep31083 |
| KT726036.1 | DR098 | Shigatse Prefecture | 10.1038/srep31083 |
| KT725986.1 | DR030 | Shigatse Prefecture | 10.1038/srep31083 |
| KT725969.1 | DR013 | Shigatse Prefecture | 10.1038/srep31083 |
| KT725990.1 | DR034 | Shigatse Prefecture | 10.1038/srep31083 |
| KT726043.1 | DR109 | Shigatse Prefecture | 10.1038/srep31083 |
| KT726009.1 | DR058 | Shigatse Prefecture | 10.1038/srep31083 |
| KT725963.1 | DR006 | Shigatse Prefecture | 10.1038/srep31083 |
| KT726028.1 | DR084 | Shigatse Prefecture | 10.1038/srep31083 |
| KT725959.1 | DR001 | Shigatse Prefecture | 10.1038/srep31083 |
| KT726014.1 | DR070 | Shigatse Prefecture | 10.1038/srep31083 |
| KT725962.1 | DR005 | Shigatse Prefecture | 10.1038/srep31083 |
| KT725997.1 | DR042 | Shigatse Prefecture | 10.1038/srep31083 |
| KT725961.1 | DR004 | Shigatse Prefecture | 10.1038/srep31083 |
| KT725982.1 | DR026 | Shigatse Prefecture | 10.1038/srep31083 |
| KT726041.1 | DR105 | Shigatse Prefecture | 10.1038/srep31083 |
| KT726030.1 | DR086 | Shigatse Prefecture | 10.1038/srep31083 |
| KT726023.1 | DR079 | Shigatse Prefecture | 10.1038/srep31083 |
| KT726013.1 | DR069 | Shigatse Prefecture | 10.1038/srep31083 |
| KT726021.1 | DR077 | Shigatse Prefecture | 10.1038/srep31083 |
| KT725994.1 | DR039 | Shigatse Prefecture | 10.1038/srep31083 |
| KT725992.1 | DR037 | Shigatse Prefecture | 10.1038/srep31083 |
| KT725971.1 | DR015 | Shigatse Prefecture | 10.1038/srep31083 |
| KT725970.1 | DR014 | Shigatse Prefecture | 10.1038/srep31083 |
| KT726024.1 | DR080 | Shigatse Prefecture | 10.1038/srep31083 |
| KT725978.1 | DR022 | Shigatse Prefecture | 10.1038/srep31083 |
| KT725984.1 | DR028 | Shigatse Prefecture | 10.1038/srep31083 |
| KT726044.1 | DR110 | Shigatse Prefecture | 10.1038/srep31083 |
| KT726003.1 | DR048 | Shigatse Prefecture | 10.1038/srep31083 |
| KT725972.1 | DR016 | Shigatse Prefecture | 10.1038/srep31083 |
| KT725960.1 | DR002 | Shigatse Prefecture | 10.1038/srep31083 |
| KT726019.1 | DR075 | Shigatse Prefecture | 10.1038/srep31083 |
| KT725987.1 | DR031 | Shigatse Prefecture | 10.1038/srep31083 |
| KT726027.1 | DR083 | Shigatse Prefecture | 10.1038/srep31083 |
| KT726038.1 | DR101 | Shigatse Prefecture | 10.1038/srep31083 |
| KT726004.1 | DR049 | Shigatse Prefecture | 10.1038/srep31083 |
| KT725985.1 | DR029 | Shigatse Prefecture | 10.1038/srep31083 |
| KT725977.1 | DR021 | Shigatse Prefecture | 10.1038/srep31083 |
| KT726000.1 | DR045 | Shigatse Prefecture | 10.1038/srep31083 |
| KT726016.1 | DR072 | Shigatse Prefecture | 10.1038/srep31083 |
| KT726025.1 | DR081 | Shigatse Prefecture | 10.1038/srep31083 |
| KT725995.1 | DR040 | Shigatse Prefecture | 10.1038/srep31083 |
| KT726010.1 | DR060 | Shigatse Prefecture | 10.1038/srep31083 |
| KT726011.1 | DR062 | Shigatse Prefecture | 10.1038/srep31083 |
| KT726012.1 | DR066 | Shigatse Prefecture | 10.1038/srep31083 |
| KT725976.1 | DR020 | Shigatse Prefecture | 10.1038/srep31083 |
| KT726029.1 | DR085 | Shigatse Prefecture | 10.1038/srep31083 |
| MF737342.1 | -- | Xinjiang Uygur | -- |
| KT725980.1 | DR024 | Shigatse Prefecture | 10.1038/srep31083 |
| KT726015.1 | DR071 | Shigatse Prefecture | 10.1038/srep31083 |
| KT726026.1 | DR082 | Shigatse Prefecture | 10.1038/srep31083 |
| KT725973.1 | DR017 | Shigatse Prefecture | 10.1038/srep31083 |
| KT726022.1 | DR078 | Shigatse Prefecture | 10.1038/srep31083 |
| KT725996.1 | DR041 | Shigatse Prefecture | 10.1038/srep31083 |
| KT725991.1 | DR036 | Shigatse Prefecture | 10.1038/srep31083 |
| KT726006.1 | DR051 | Shigatse Prefecture | 10.1038/srep31083 |
| KT725964.1 | DR007 | Shigatse Prefecture | 10.1038/srep31083 |
| KT725968.1 | DR012 | Shigatse Prefecture | 10.1038/srep31083 |
| KT725974.1 | DR018 | Shigatse Prefecture | 10.1038/srep31083 |
| KT726040.1 | DR103 | Shigatse Prefecture | 10.1038/srep31083 |
| KT725965.1 | DR008 | Shigatse Prefecture | 10.1038/srep31083 |
| KT726005.1 | DR050 | Shigatse Prefecture | 10.1038/srep31083 |
| KT725988.1 | DR032 | Shigatse Prefecture | 10.1038/srep31083 |
| KT725981.1 | DR025 | Shigatse Prefecture | 10.1038/srep31083 |
| KT725967.1 | DR010 | Shigatse Prefecture | 10.1038/srep31083 |
| KT726034.1 | DR092 | Shigatse Prefecture | 10.1038/srep31083 |
| KT726017.1 | DR073 | Shigatse Prefecture | 10.1038/srep31083 |
| KT725979.1 | DR023 | Shigatse Prefecture | 10.1038/srep31083 |
| KT726020.1 | DR076 | Shigatse Prefecture | 10.1038/srep31083 |
| KT726018.1 | DR074 | Shigatse Prefecture | 10.1038/srep31083 |
| KT726035.1 | DR094 | Shigatse Prefecture | 10.1038/srep31083 |
| KT725999.1 | DR044 | Shigatse Prefecture | 10.1038/srep31083 |
| KT725983.1 | DR027 | Shigatse Prefecture | 10.1038/srep31083 |
| KT726001.1 | DR046 | Shigatse Prefecture | 10.1038/srep31083 |
| KT726008.1 | DR055 | Shigatse Prefecture | 10.1038/srep31083 |
| KT726032.1 | DR088 | Shigatse Prefecture | 10.1038/srep31083 |
| KT726031.1 | DR087 | Shigatse Prefecture | 10.1038/srep31083 |
| KT726042.1 | DR106 | Shigatse Prefecture | 10.1038/srep31083 |
| KT726033.1 | DR090 | Shigatse Prefecture | 10.1038/srep31083 |
| KT726039.1 | DR102 | Shigatse Prefecture | 10.1038/srep31083 |
| KT725998.1 | DR043 | Shigatse Prefecture | 10.1038/srep31083 |
